# Widespread Contamination by Anticoagulant Rodenticides in Insectivorous Wildlife from the Canary Islands: Exploring Alternative Routes of Exposure

**DOI:** 10.3390/toxics13060505

**Published:** 2025-06-15

**Authors:** Beatriz Martín Cruz, Andrea Acosta Dacal, Ana Macías-Montes, Cristian Rial-Berriel, Manuel Zumbado, Luis Alberto Henríquez-Hernández, Ramón Gallo-Barneto, Miguel Ángel Cabrera-Pérez, Octavio P. Luzardo

**Affiliations:** 1 Toxicology Unit, Research Institute of Biomedical and Health Sciences (IUIBS), Universidad de Las Palmas de Gran Canaria, Paseo Blas Cabrera s/n, 35016 Las Palmas de Gran Canaria, Spain; andrea.acosta@ulpgc.es (A.A.D.); ana.macias@ulpgc.es (A.M.-M.); cristian.rial@ulpgc.es (C.R.-B.); manuel.zumbado@ulpgc.es (M.Z.); luis.henriquez@ulpgc.es (L.A.H.-H.); octavio.perez@ulpgc.es (O.P.L.); 2 Spanish Biomedical Research Center in Physiopathology of Obesity and Nutrition (CIBERObn), Avenida Monforte de Lemos 3-5, Pabellón 11, Planta 0, 28029 Madrid, Spain; 3 Gestión y Planeamiento Territorial y Medioambiental, S.A. (GESPLAN), Canary Islands Government, C/León y Castillo 54, bajo, 35003 Las Palmas de Gran Canaria, Spain; 4 General Directorate to Combat Climate Change and the Environment, Biodiversity Service, Canary Islands Government, Plaza de los Derechos Humanos, 22, Edificio Servicios Múltiples I. 8ª Planta, 35071 Las Palmas de Gran Canaria, Spain

**Keywords:** insects, biomonitoring, food chain, brodifacoum, non-raptor birds, reptiles

## Abstract

Research on anticoagulant rodenticides (ARs) in wildlife has primarily focused on apex predators, with less attention given to their potential integration into lower trophic levels and the associated exposure pathways. At the base of the terrestrial food web, invertebrates have been suggested as potential vectors of ARs to insectivorous species such as small mammals, reptiles, and birds. To explore this hypothesis, we analyzed the presence of nine anticoagulant rodenticides—including both first-generation (FGARs) and second-generation (SGARs) rodenticides—in 36 liver samples from Yemen chameleons (*Chamaeleo calyptratus*) and 98 liver samples from six non-raptorial, predominantly insectivorous bird species from the Canary Islands. Through HPLC-MS/MS analysis, only SGARs were detected in both animal groups collected between 2021 and 2024. Approximately 80% of reptiles and 40% of birds tested positive for at least one SGAR, with brodifacoum being the most frequently detected compound. In more than 90% of positive cases, it was found as the sole contaminant, while co-occurrence with other SGARs was uncommon. Additionally, most concentrations were below 50 ng/g wet weight, except for two bird specimens, suggesting heterogeneous exposure scenarios and potential variability in contamination sources across individuals. These findings provide evidence of AR integration at the base of the terrestrial food web in the Canary Islands and suggest secondary exposure via invertebrates as a plausible route of contamination. Further research directly analyzing invertebrate samples is needed to confirm their role as vectors of ARs to insectivorous wildlife in insular ecosystems.

## 1. Introduction

Anticoagulant rodenticides (ARs), particularly second-generation compounds (SGARs), are widely used across urban and agricultural environments to control rodent populations [1,2]. Although highly effective, these substances pose substantial risks to non-target wildlife due to their high toxicity, environmental persistence, and bioaccumulation [3,4]. SGARs inhibit vitamin K epoxide reductase (VKORC), a key enzyme in the blood clotting cascade, causing fatal hemorrhaging in exposed animals [5,6]. Beyond lethal effects, there is growing concern over sublethal effects on the health and behavior of non-target species [7,8,9,10], prompting regulatory efforts to mitigate their impact—although these measures have shown limited success [3,11,12,13,14].

Most research to date has focused on top predators—such as birds of prey, scavengers [13,15,16,17,18,19], and carnivorous mammals [20,21,22]—due to their trophic position and high susceptibility to secondary poisoning. However, knowledge on how SGARs affect lower trophic levels remains limited, despite the essential ecological roles of these species [23]. While direct consumption of bait (primary exposure) and ingestion of contaminated prey (secondary exposure) are well-documented for target species and apex predators, the exposure of non-target prey species—such as small mammals, reptiles, and non-raptorial birds—remains underexplored [24,25,26]. These animals may consume bait directly due to its attractive formulation or indirectly through ingestion of contaminated invertebrates, plants, soil, or even water sources [10,25,27,28,29,30].

Factors such as habitat, diet, and foraging behavior play key roles in shaping AR exposure risk across trophic groups [31,32]. Insectivorous predators, in particular, may represent an interesting secondary exposure route, given the potential role of invertebrates as AR vectors. Experimental studies have shown that various invertebrate species can consume rodenticide bait without exhibiting acute toxicity [33,34,35]—likely due to fundamental differences in their hemostatic systems compared to vertebrates [36]. However emerging evidence indicates that invertebrates may also experience toxic or sublethal effects [37,38,39]. Likewise, field observations from island eradication campaigns and rodenticide-treated agricultural areas have documented invertebrates feeding on bait, rodent feces, and animal carcasses [10,25,40,41,42], further supporting their potential role as contamination vectors.

Their capacity to accumulate residues, coupled with apparent physiological resilience, raises significant concerns about trophic transfer to insectivorous vertebrates. This indirect pathway has been proposed in mammals [43,44], reptiles [32,45], and birds [31,46], although causal links between AR residues in invertebrates and predator exposure remain unclear. In the Canary Islands, recent studies confirm the widespread presence of SGARs in top predators, both native and invasive, suggesting broad environmental contamination [11,47,48,49,50,51]. However, biomonitoring efforts targeting lower trophic levels—particularly non-raptorial birds and reptiles—are virtually nonexistent.

Intentional poisoning has been reported in some endemic reptile species within the Canary Islands [47,49], and the invasive California kingsnake (*Lampropeltis californiae*) has been proposed as a potential sentinel species for AR exposure due to its predatory habits and position at the top of the island food chain [48]. Nevertheless, no previous studies have assessed AR exposure in insectivorous reptiles. For this purpose, we selected the veiled chameleon (*Chamaeleo calyptratus*), an invasive species with an insectivorous diet [52,53], as a sentinel candidate. Its trophic position closely mirrors that of several protected endemic reptiles such as *Gallotia stehlini*, *Chalcides sexlineatus*, and *Tarentola boettgeri* [54,55,56,57]—for which biomonitoring is challenging due to their high conservation status in the archipelago.

Similarly, research on AR exposure in Canary Island birds has primarily focused on raptors [11,47,49,50,51], reporting both high prevalence and alarming residue concentrations in species such as the Eurasian kestrel (*Falco tinnunculus*) [11,51]. However, studies in non-raptorial birds are scarce and typically limited to cases of confirmed poisoning [47]. To date, no investigations have evaluated AR exposure in non-raptors from an ecotoxicological perspective, particularly in relation to dietary habits. To address this gap, we evaluated six non-raptorial bird species—some of which are endemic subspecies of the Canary Islands (*Upupa epops*, *Burhinus oedicnemus*, *Chlamydotis undulata*, *Turdus merula*, *Dendrocopos major*, *Apus apus*)—whose diets consist primarily or substantially of invertebrates [58,59,60,61,62,63,64,65,66,67]. These species could offer a valuable perspective on the potential exposure of insectivorous predators within the archipelago.

Based on the hypothesis that invertebrates could act as vectors for ARs in insectivorous species, the aims of this study were as follows:

(i) Assess the integration of ARs into lower trophic levels of the Canary Islands’ terrestrial ecosystem by analyzing liver samples from six insectivorous non-raptor bird species and one invasive insectivorous reptile (*Chamaeleo calyptratus*);
(ii) Evaluate the potential role of invertebrates as vectors of ARs for these lower-level predators.

## 2. Materials and Methods

### 2.1. Study Area, Sampling, and Ethical Statements

This study was conducted in the Canary Islands, an archipelago of eight inhabited islands and several islets located in the Atlantic Ocean and part of the Natura 2000 network within the Macaronesian biogeographic region [68]. Covering approximately 7000 km^2^ and home to around 2.2 million people [69], the archipelago includes 154 Sites of Community Importance (SCIs) and 45 Special Protection Areas for Birds (SPAs) [70]. The Canary Islands are considered one of Europe’s biodiversity hotspots, hosting a high proportion of endemic species—about 45% of the fauna and 25% of the flora [68]. In contrast, 48 species are currently classified as invasive alien species of concern in the region [71].

To contribute to the conservation of native biodiversity, liver samples were collected between 2021 and 2024 during necropsies of 36 Veiled chameleons (*Chamaeleo calyptratus*) and 98 non-raptorial birds: 22 endemic Canary bustards (*Chlamydotis undulata fuertaventurae*), 44 stone-curlews (*Burhinus oedicnemus insularum*, endemic to Fuerteventura, Lanzarote, and La Graciosa, and *B. oedicnemus distinctus* in the rest of the archipelago), 5 hoopoes (*Upupa epops*), 5 woodpeckers (*Dendrocopos major canariensis*, endemic to Tenerife, and *D. major thanneri*, endemic to Gran Canaria), 4 common swifts (*Apus apus*), and 18 blackbirds (*Turdus merula*).

Bird samples were collected from five of the eight islands (Fuerteventura, Gran Canaria, Tenerife, Lanzarote, and La Palma), while all chameleon samples were collected from Gran Canaria, where this species has established. No animals were sacrificed for the purpose of this study. Instead, bird specimens were either found dead in the wild, died during veterinary care, or were humanely euthanized by veterinarians due to irreversible conditions incompatible with recovery. The chameleons were captured and euthanized within the framework of an eradication program, as this species is officially recognized as an invasive alien species of concern in the outermost regions of the Canary Islands [72]. All necropsies were performed by veterinary personnel, and both carcasses and extracted livers were stored at –20 °C until toxicological analysis. Chemical analyses were conducted at the Toxicology Service (SERTOX) of the University of Las Palmas de Gran Canaria as part of the toxicological monitoring efforts coordinated by the Canary Islands Wildlife Health Surveillance Network (Red VIGIA) [73], aimed at assessing environmental threats to wildlife health.

Biological data for chameleons were recorded during necropsy, and geolocation data were provided by personnel from the eradication program. Recorded variables included sex, body weight, and snout-vent length (SVL). All chameleons were collected in Gran Canaria (Arucas municipality). Of the 36 individuals analyzed, 38.8% were female (n = 14), 55.6% were male (n = 20), and 5.6% were undetermined due to underdeveloped sexual traits (n = 2) (Table S2). No macroscopic signs of coagulopathy consistent with rodenticide poisoning were observed during necropsy.

Biological and geographical data were incomplete for all bird specimens due to variable preservation status and diverse origins. For those with available information, geographic data were restricted to the island of origin. The sex ratio was nearly balanced (n = 21 females, n = 20 males), with adults representing the most frequent age group (n = 33, 33.7%). Most individuals were in optimal body condition (n = 32, 32.7%). Trauma, including collisions with vehicles, power lines, wind turbines, or unspecified traumatic events, was the confirmed cause of death in 33.7% of cases. Natural causes, such as infectious or parasitic diseases, accounted for 15.3% of deaths (n = 15). Rodenticide poisoning was not identified as the direct cause of death in any specimen.

### 2.2. Analysis of Anticoagulant Rodenticides in Liver Tissue and Sample Preparation

Anticoagulant rodenticides were analyzed using certified standards and internal procedural standards (P-IS, (±)-Warfarin-d5) of maximum purity (98–99.8%) from Dr. Ehren-Storfer^®^ (Augsburg, Germany). Among the ARs included in the panel, four were first-generation (chlorophacinone, coumachlor, coumatetralyl, and diphacinone), and five were second-generation (brodifacoum, bromadiolone, difenacoum, difethialone, and flocoumafen). As for solvents, acetonitrile, methanol (ACN and MeOH, >99.9% purity), and formic acid (FA, 98% purity), all LC-MS grade and from Honeywell^®^ (Morristown, NJ, USA), were used. Additionally, the water required for the analyses was produced in-house using a MilliQ^®^ A10 system (Millipore, Molsheim, France). The QuEChERS extraction salts, AOAC method (6 g of magnesium sulfate and 1.5 g of sodium acetate), were obtained from Agilent Technologies^®^ (Palo Alto, CA, USA).

To ensure accurate analysis, quality control (QC) samples were incorporated in the series at a concentration of 5 ng/g, ensuring relative standard deviation (RSD) values ≤20% and recovery (REC) rates between 70–120%. In addition, a ten-point calibration curve was prepared using a blank chicken liver matrix, covering concentrations from 0.195 to 100 ng/g. Prior to extraction, all samples, calibration curve, blanks, and QCs were fortified with the P-IS solution, and the resulting concentrations were reported on a wet weight (ww) basis.

The extraction of analytes from samples, QCs, and calibration curve was performed following the method described by our research team [74,75]. The extraction process was carried out using a micro-QuEChERS approach, as follows. For this procedure, 1 g of liver was homogenized using a Precellys Evolution^®^ system (Bertin Technologies, Rockville, Washington D.C., USA). Subsequently, the homogenized matrix was diluted with 4 mL of ultrapure water, and 1 g of this solution was transferred to a 5 mL Eppendorf^®^ tube, to which 20 μL of P-IS at 100 ppb and 2 mL of ACN (0.5% FA) were added. Then, the solution was subjected to ultrasound (VWR^®^, 50/60 Hz, 120 W), and 480 mg of magnesium sulfate and 120 mg of sodium acetate were added to each sample tube, vortexed, and manually shaken. Finally, they were centrifuged at 4200 rpm and 2 °C for 15 min (5804 R, Eppendorf, Hamburg, Germany), and the supernatant was filtered through a Chromafil^®^ PET-20/15 filter with a 0.2 μm pore size (Macherey-Nagel, Düren, Germany).

Finally, quantitative analysis was performed using an Agilent 1290 UHPLC system (Agilent Technologies, Palo Alto, CA, USA) coupled with an Agilent 6460 triple quadrupole mass spectrometer. The chromatographic configuration incorporated a heated InfinityLab Poroshell 120 column, equipped with an inline filter and a UHPLC guard column. A gradient elution method was applied, using a mobile phase composed of 0.1% formic acid and 2 mM ammonium acetate in water (Phase A) and 2 mM ammonium acetate in methanol (Phase B). The injection volume was set to 8 μL, with a flow rate of 0.4 mL/min. The mass spectrometer operated in dynamic multiple reaction monitoring (dMRM) mode, covering both positive and negative ionization polarities, with precisely defined cycle, dwell, and run times. Additional operational parameters for the Agilent Jet Stream Electrospray Ionization Source (AJS-ESI) and the gas settings used are detailed in the referenced methodologies, which also include the parameters for method validation [74,76].

### 2.3. Statistical Analyses

To perform descriptive statistical analyses, JAMOVI^®^ v.2.4.7 (The Jamovi Project, 2023; available at https://www.jamovi.org (accessed on 9 June 2025)) was employed.

Descriptive data were expressed as mean, medians, standard deviation, and interquartile ranges (25th–75th percentiles). Additionally, detection frequency was determined as the percentage of animals with at least one SGAR detected in their liver tissues. Animals with concentrations above the limit of quantification (LOQ), as well as those with concentrations below the LOQ but above the limit of detection (LOD), were considered as positive detections; in the latter case, random values within the LOQ–LOD range (Table S1) were assigned. Concentrations below the LOD were classified as non-detected and assigned a random value between zero and half the LOD for statistical analysis purposes.

The graphical resources and illustrations were created using BioRender and Infogram.

## 3. Results and Discussion

Biomonitoring of anticoagulant rodenticides (ARs) has primarily focused on top predators [13,15,16,17,18,19,20,21,22], while exposure routes in prey species like insectivorous reptiles and birds have received less attention [23]. However, recent evidence suggests that ARs may also affect insectivores, possibly through invertebrate-mediated exposure [31,32,43,44,45,46]. To explore this, we analyzed liver samples from veiled chameleons (*Chamaeleo calyptratus*) and six non-raptorial bird species with predominantly insectivorous diets in the Canary Islands for nine AR compounds, including first-generation rodenticides (FGARs: coumatetralyl, diphacinone, warfarin, and chlorophacinone) and second-generation rodenticides (SGARs: brodifacoum, bromadiolone, difenacoum, flocoumafen, and difethialone).

### 3.1. SGARs Exposure in Reptiles: Veiled Chamaeleon as a Case Study

Although the study of anticoagulant rodenticides (ARs) in reptiles has historically been limited compared to other taxa, several authors have begun to recognize both their direct and indirect interactions with these compounds, as well as their potential role as vectors [32,45].

**Figure 1:**
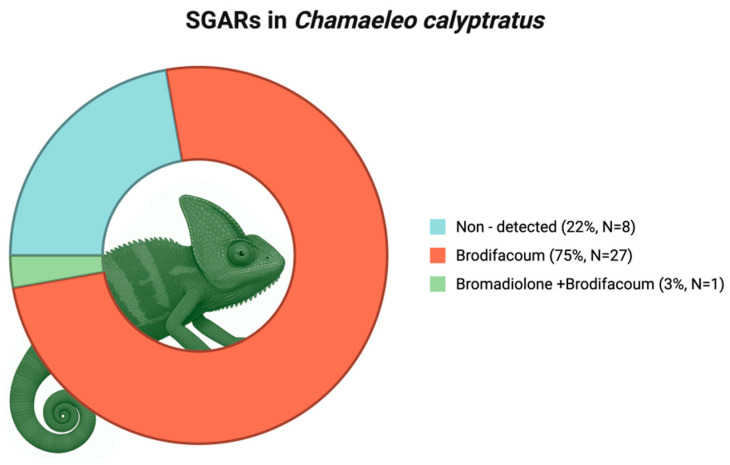
Donut chart showing the proportion and sample size of *Chamaeleo calyptratus* individuals analyzed for anticoagulant rodenticides (ARs), differentiating between specimens with non-detected compounds and those that tested positive. Created in BioRender: https://BioRender.com/dmusrqq (accessed on 9 June 2025).

In the present study, the livers of 36 individuals of veiled chameleon (*Chamaeleo calyptratus*), an arboreal insectivore reptile, were analyzed [52,53]. Of the nine ARs investigated, including both SGARs and FGARs, only two SGARs were detected: brodifacoum and bromadiolone. In total, 77.8% (n = 28) of the chameleons tested positive, suggesting their potential use as a sentinel species at this trophic level. Brodifacoum was the predominant compound, found in all the positive individuals (n = 28), reaching a maximum concentration of 33.11 ng/g wet weight (ww) (Table 1, Figure 1). Bromadiolone, on the other hand, was detected in only one individual, in combination with brodifacoum, at 0.47 ng/g ww.

These frequencies partially align with a previous study in the Canary Islands, which found brodifacoum in 62.7% (n = 23) of giant lizards *(Gallotia gallotia)* [47]. However, the median concentrations were much higher in the lizards (562.3 ng/g ww.) compared to the chameleons (3.13 ng/g ww.), possibly due to their association with intentional poisoning events, leading to a bias toward higher levels. In both studies, brodifacoum was the only compound detected, except for one isolated case of bromadiolone in a chameleon, reinforcing the prevalence of this compound as the main rodenticide used on the islands [11,48,51]. ARs have also been linked to poisoning incidents in other endemic reptiles of the Canary Islands, such as the skink (*Chalcides simonyi*, n = 1), although concentrations were not detailed [49]. Furthermore, a recent study in Gran Canaria by our team identified high exposure to ARs in the invasive and generalist predator California kingsnake (*Lampropeltis californiae*). Over 90% of the analyzed specimens contained ARs, with brodifacoum as the most frequently observed compound. In contrast to chameleons, up to five different SGARs were detected in the snakes, and more than 50% were exposed to combinations of these compounds [48]. These differences could be explained by their distinct trophic positions: while chameleons are strictly insectivorous, snakes primarily feed on rodents and reptiles [77,78], which increases their risk of exposure, as also suggested by other authors [32,79].

In other regions of Spain, a positive case of flocoumafen was reported in a horseshoe whip snake (*Hemorrhois hippocrepis*), linked to the use of this compound during a seabird protection campaign in the Chafarinas Islands [80]. However, no ARs were found in the sole individual of *Testudo hermanni* in Aragón [81]. Internationally, three species of urban reptiles were evaluated in Australia, observing varying levels of exposure based on diet and trophic level. They reported 91% in rodent-predating snakes, 60% in omnivorous dugites, and 45% in tiger snakes (amphibian specialists) [32]. In our study, 77.8% of the chameleons, captured in an urban environment, tested positive, representing an intermediate value, which is relevant given their predominantly insectivorous diet. However, the average concentration in chameleons (6.83 ng/g ww) was lower than that of the Australian species at 178 ng/g, 40 ng/g, and 9 ng/g, respectively. This may be related to differences in trophic behavior: while in Australia, direct consumption of baits and garbage has been observed, the chameleons in Gran Canaria have not shown such anthropogenic interactions. However, consistent with our findings, brodifacoum was identified as the most prevalent compound, along with bromadiolone and difenacoum [32].

In contrast, other authors analyzed 185 pit vipers and 89 green anoles on a Pacific island, detecting only FGARs (warfarin, diphacinone, and coumatetralyl). The prevalence was 9% in pit vipers (max. 436.5 ng/g), and there was only one positive case in anoles (51.9 ng/g diphacinone) [79]. In comparison, the chameleons in our study showed similar maximum concentrations to the anoles with a similar diet but with a much higher prevalence. The exclusive detection of FGARs could be due to the compound used in each area, with diphacinone being the reference product on that island in previous years.

Regarding toxicological risk, there are no established toxicity thresholds for reptiles. However, lower susceptibility compared to birds and mammals has been suggested, along with a possible capacity for tolerance and biomagnification [42,45,82]. The concentrations observed in the chameleons (<50 ng/g ww) are considered to have a low probability of toxicity in other vertebrates [83]. Nevertheless, information on this species is scarce, and susceptibility may vary significantly among reptiles. Apparently, resistance has been suggested in lava lizards [84], geckos [27], monitors [85], boas, and tortoises [86], which were exposed to rodenticides through eradication programs and/or laboratory experiments, and they rarely experienced mortality. However, other authors suggest greater sensitivity in species such as green turtles [87], iguanas, and ameivas [86].

Finally, it is worth noting that all the chameleons were alive at the time of capture and showed no macroscopic signs of coagulopathy, consistent with other studies [79,86]. No direct interaction with baits has been documented either, suggesting that insects may act as vectors, as proposed by other authors [32,45]. As a result, endemic insectivorous species in the Canary Islands, such as *Gallotia stehlini*, *Chalcides sexlineatus*, and *Tarentola boettgeri*, may also be at risk of exposure. Further studies should include stomach content analysis and should expand to other reptile species in the archipelago to better assess the real threat that ARs pose to the local herpetofauna.

### 3.2. SGARs Exposure in Non-Raptor Birds: Insectivorous Birds

Non-raptor birds may be exposed to anticoagulant rodenticides (ARs) through primary or secondary pathways [26,28,88,89]. Over 600 incidents of rodenticide poisoning have been documented across 15 countries, with exposure levels influenced by species’ diets, foraging behavior, and even morphology [31]. Granivorous birds are categorized at high risk of primary exposure, while insectivorous birds are classified as having a moderate risk of secondary exposure to ARs [26,82].

**Figure 2:**
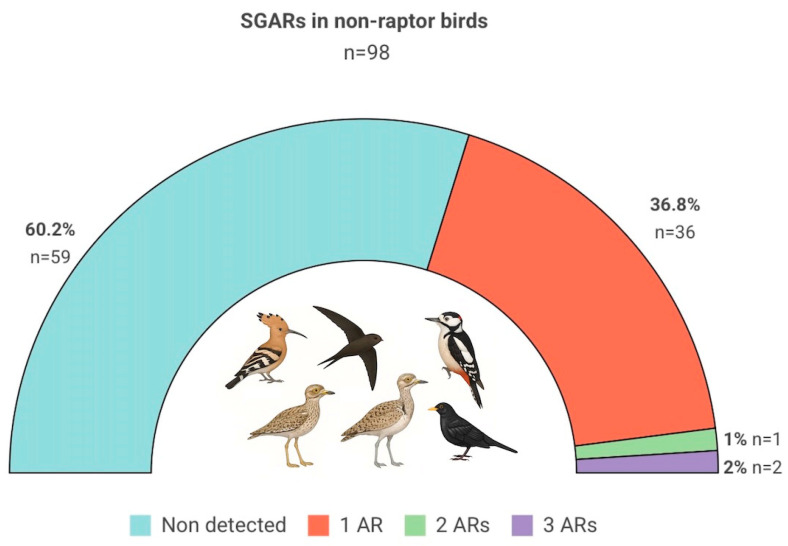
Semi-circle chart showing the proportion and sample size of six non-raptorial bird species—hoopoe (*Upupa epops*), stone-curlew (*Burhinus oedicnemus*), houbara bustard (*Chlamydotis undulata*), blackbird (*Turdus merula*), woodpecker (*Dendrocopos major*), and common swift (*Apus apus*)—analyzed for anticoagulant rodenticides (ARs), distinguishing between individuals with no detected compounds and those testing positive for one or more second-generation anticoagulant rodenticides (SGARs). Created in BioRender. https://BioRender.com/i16zaee (accessed on 9 June 2025).

In this study, we analyzed six species of non-raptor birds with diets consisting predominantly or substantially of insects [58,59,60,61,62,63,64,65,66,67]. These species differ in habitat use, foraging strategies, and morphology. Of the total birds analyzed (n = 98), 39.8% (n = 39) tested positive for at least one SGAR, with no detection of first-generation compounds (Figure 2). Among the SGARs detected—brodifacoum, bromadiolone, difenacoum, and difethialone—brodifacoum was the most detected, found in 92.3% (n = 36) of positive individuals, with a maximum concentration of 451.2 ng/g ww. It was generally detected alone (91.6%, n = 33), but it was also present in combination with other SGARs in three individuals (brodifacoum + bromadiolone, n = 1; brodifacoum + bromadiolone + difethialone, n = 2). Bromadiolone (n = 2) and difenacoum (n = 1) were also detected individually, at lower concentrations (1.35 ng/g ww, 0.87 ng/g ww and 3.16 ng/g ww, respectively). Difethialone was only detected in two individuals (11.9 ng/g ww and 2.94 ng/g ww), both in combination with other SGARs (Table S3).

Although there are no previous studies focused exclusively on insectivorous birds in the Canary Islands, AR exposure in non-raptor birds has been previously documented. However, many of these cases were linked to intentional poisoning incidents [47]. In a previous study involving 343 birds, 4.7% (n = 16) tested positive, with the highest exposure found in the common raven (n = 7/97, 7.2%). Additional positive detections were reported in grey herons (n = 2), stone-curlews (n = 3), European turtle doves (n = 2), Eurasian blackbirds (n = 2), and one red-legged partridge (n = 1) in another insular study [49]. While these species differ from those included in our sample, the overall detection rate was significantly lower. In contrast, our results are more comparable to those reported elsewhere, with detection rates of 52.1% in non-raptor birds [23] and 28% in passerines, with the highest rates reported in insectivores such as the European robin (44.4%, n = 22) [28]. In addition, other studies conducted in Spain documented eight positive cases in non-raptor birds [81] and found a 50.7% detection rate in granivorous birds, although many of the animals included in that study also showed signs of intentional poisoning [80].

Regarding the pattern of SGARs detected, the results of our study mirror those reported for non-raptor birds in the Canary Islands and mainland Spain, with brodifacoum, bromadiolone, difenacoum, and flocoumafen being the most detected compounds [47,81]. Similar detection patterns have been reported in raptors from the archipelago [11,47,48,51]. However, the prevalence of brodifacoum in our study (36.7%) is approximately ten times higher than previous findings in non-raptor birds from the Canary Islands at 3.9% [47]. The increase in brodifacoum detection over time has been previously observed in kestrels in Tenerife, highlighting both its persistence and the limited effectiveness of regulatory measures [11]. In contrast to a previous study that reported widespread detection of chlorophacinone (n = 71) in granivorous birds during a vole outbreak control campaign in 2007, our study did not detect any first-generation ARs [80]. However, their study also recorded brodifacoum (n = 3), bromadiolone (n = 3), and flocoumafen (n = 1), confirming the environmental persistence of SGARs even after their use had discontinued.

Likewise, only 3% of birds in our study were exposed to multiple SGARs (Figure 2), a much lower proportion than reported in raptors from the Canary Islands, where >50% carry multiple residues [11,48,51]. This difference is likely due to bioaccumulation and biomagnification in apex predators. Additionally, only two birds in our sample exceeded the 100–200 ng/g ww thresholds used in some raptors for assessing toxicity or sublethal effects [83,90,91]: one Eurasian hoopoe (451.2 ng/g ww) and one stone-curlew (235.47 ng/g ww). These values approach the maximum concentration reported in insectivorous birds, such as the dunnock (*Prunella modularis*) at 348 ng/g ww sampled in eradication areas [28]. One additional stone-curlew fell within the 50–100 ng/g ww range (50.25 ng/g ww), while the remaining individuals were below 50 ng/g ww. Importantly, none of the birds in our sample were confirmed as victims of intentional poisoning, and hemorrhages were only observed in one individual, whose cause of death was attributed to trauma. The absence of macroscopic signs of coagulopathy, even in birds with high AR levels, has been previously reported [46,80].

Additionally, the differences in concentrations observed among certain individuals may be attributed to distinct exposure pathways. The two individuals with the highest concentrations may have been exposed via primary ingestion of bait, as seen in sparrows (0.073 µg/g brodifacoum) [25] or in *Petroica australis*, where 50% of pairs were exposed to coumatetralyl after rat eradication [88]. However, it is important to recognize that this risk is much higher in granivorous birds. In contrast, the lower residual concentrations observed in most birds suggest secondary exposure through consumption of contaminated invertebrates [28]. Nevertheless, high concentrations have also been reported in nestling robins exposed secondarily through parental feeding on contaminated invertebrates (0.08 ± 0.02 µg/g brodifacoum) [46]. Mortality associated with coagulopathy has also been documented in the poouli, which consumes snails in areas where chlorophacinone was used [33]. Moreover, brodifacoum has been detected in shorebirds found dead after eradication programs, suggesting potential secondary exposure [27]. However, due to the lack of species-specific toxicity reference values for many bird species, it remains difficult to draw firm conclusions about toxicological risk.

#### Bird Exposure to Anticoagulant Rodenticides: Foraging and Habitat Considerations

As previously described, factors such as diet, habitat, and foraging behavior may influence the risk of exposure to these compounds across different trophic groups [31,32]. In our study, detection rates were highest on the two most urbanized islands (Gran Canaria: 46.2%, and Tenerife: 57.1%; Table S3), consistent with studies showing positive correlations between urban development and AR exposure [24,81,92]. However, our sample size and lack of geolocation data prevent robust spatial analyses. Future studies incorporating precise location data could provide more insight into urban–rural exposure gradients.

**Figure 3:**
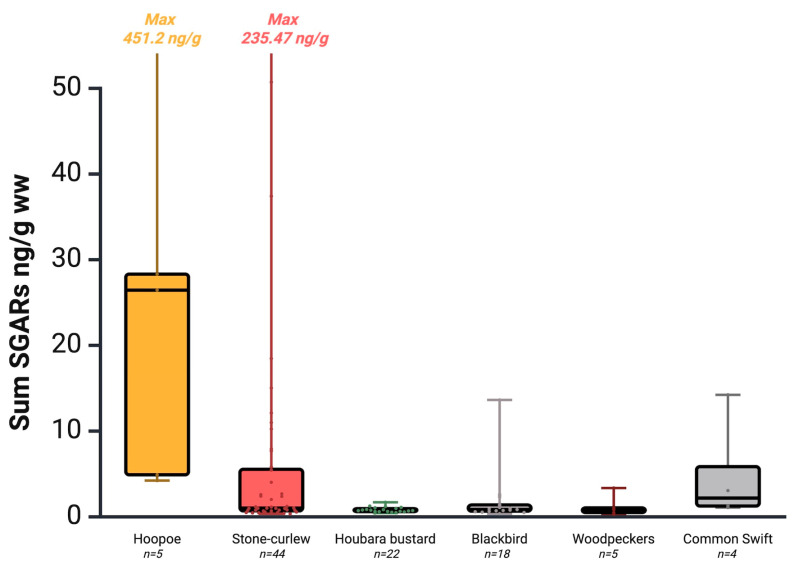
Box and whisker plot showing the comparison of ΣSGARs between six non-raptor birds (hoopoe, stone-curlew, houbara bustard, blackbird, woodpecker, and common swift). The lines represent the medians, the boxes represent the 25th to 75th percentiles, and the minimal and maximal values are shown at the ends of the bars with lines or text. Created in https://BioRender.com (accessed on 9 June 2025).

Among species, the houbara bustard was the least exposed (Table 2 and Figure 3), likely due to its preference for arid and steppe habitats—many within protected areas on Lanzarote, Fuerteventura, and La Graciosa—far from urban or intensive agricultural zones [62,93]. Similarly, the Canary woodpecker (n = 1, 20%) also showed low exposure, probably due to its restricted distribution in pine forests on Gran Canaria and Tenerife far from anthropogenic activities [66,67]. However, the sample representation of this species is too limited to draw conclusions.

In contrast, all hoopoes (n = 5) tested positive, including the individual with the highest concentration (451.2 ng/g ww.). This may be linked to their foraging behavior of insects on bare ground [58,59,94], a trait associated with higher AR exposure risk [31]. Nonetheless, the small sample size precludes firm conclusions. Stone-curlews and blackbirds were the following most exposed species, and they have been previously reported in the islands [47]. Exposure in stone-curlews could be related to their broad surface-feeding behavior and preference for traditional agricultural landscapes, generally abandoned, and semi-natural grassland [64,95]. Likewise, their potential interaction with agricultural areas has also been linked to higher exposure levels in other animals [24,96]. Similarly, the fact that the blackbird is one of Europe’s most common urban birds [97] may also contribute to its AR exposure, as habituation to human presence and the possible opportunistic consumption of novel food items have been suggested as potential risk factors [31]. In this species, the role of diet, based on insects and earthworms, in relation to heavy metal contamination has already been assessed, suggesting that the influence of landscape on pollutant transfer may be related to dietary variations [65]. A similar influence could occur with other compounds such as anticoagulant rodenticides.

Finally, the detection of AR residues in swifts (n = 3, 75%) was unexpected, as these birds spend most of their lives flying and feed exclusively on aerial invertebrates. Their exposure is therefore most likely attributable to the ingestion of contaminated prey. Swifts have previously been used as bioindicators of atmospheric contamination with pesticides and PCBs, and microplastics have even been found in their digestive tracts, likely ingested via contaminated insects [60,61,98].

Overall, the individual and interspecific variability in AR exposure is consistent with previous findings, though conclusive associations remain elusive due to variation in ecology, metabolism, susceptibility, and other factors [28,31]. In our study, the limited sample sizes for many species and islands precluded robust statistical analyses. Future studies with larger, geographically stratified samples and stomach content analyses would be valuable for confirming invertebrate predation as the primary exposure route.

### 3.3. Role of Invertebrates as a Potential Vector of Anticoagulant Rodenticides

The interaction between invertebrates and anticoagulant rodenticides (ARs) has been documented both in laboratory experiments and field observations, including coastal environments following eradication campaigns [99]. Some authors have demonstrated that invertebrates are capable of directly ingesting bait [40,41,42,44], while others have provided evidence of exposure by analyzing the movement of ARs through the food web, including ingestion of feces from intoxicated small mammals, rodent carcasses, or soil-bound AR residues [10,25].

In general, invertebrates appear more resistant than vertebrates due to physiological differences in their coagulation systems [36]. Nevertheless, sublethal effects have been observed, such as increased shelter-seeking behavior and reduced activity, boldness, and aggressiveness [37]. A concentration of 1 mg/kg of bromadiolone in soil has also been reported to inhibit earthworm growth and elevate biomarkers of oxidative stress [38]. Furthermore, brodifacoum at 100 ppb has been shown to reduce fertilization success in coral gametes and larvae by up to 15%, although this concentration does not reflect a likely environmental scenario [39].

Given that ingestion is the primary route of exposure, the potential role of insects as AR vectors highlights the need to assess the risk they may pose to their predators such as mammals, reptiles, and birds, including raptors that feed on invertebrates such as kestrels, little owls, and moreporks [23]. However, additional exposure routes, such as the direct ingestion of baits, should also be considered in light of the high detection rates recorded in this study. Likewise, other factors such as foraging behavior in high-risk habitats or a more generalist diet may further influence exposure levels.

Insectivorous predators could therefore be secondarily exposed to these compounds, but the actual risk remains uncertain. Insectivorous birds have been classified as having a moderate risk and insectivorous reptiles and raptors as having a low risk of secondary exposure in Southeast Asian oil palm plantations [82]. It has been estimated that a bird would need to consume its body weight in contaminated cockroaches to reach a 50% risk of acute intoxication, suggesting a relatively low risk compared to primary exposure. The same study also reported that AR concentrations in invertebrates decline rapidly within the first two weeks and more slowly over the following four weeks [100]. However, a potential acute, repeated, or chronic risk to species such as shrews, starlings, and hedgehogs following the ingestion of contaminated slugs had been suggested [34]. Similarly, a 3–8% mortality risk and a 0.42–11% sublethal coagulopathy risk have been predicted for pooulis feeding on contaminated snails, with acceptable risk levels for quail and mallards [33].

Therefore, it is reasonable to suggest that invertebrates in the Canary Islands’ insular ecosystem may act as vectors of ARs to insectivorous predators considering diet as the primary exposure route and the concentrations found in most of the studied animals (<50 ng/g ww). However, targeted studies analyzing AR residues directly in invertebrate prey would be necessary to confirm this exposure pathway and better evaluate the potential ecological risk involved.

## 4. Limitations and Strengths

This study has several limitations that should be acknowledged. First, the overall sample size, although considerable for a wildlife toxicology investigation, remains modest—particularly within the avian group, where the number of individuals per species was relatively low. This limitation is largely attributable to the opportunistic nature of sample collection, which relied on animals received through the Canary Islands’ wildlife health surveillance system (Red Vigía) for diagnosis of suspected poisonings or post-mortem investigations. Consequently, sampling was constrained by the availability of deceased animals and did not allow for targeted collection to equalize group sizes.

Despite the limited number of individuals per species, we deliberately opted for a broader representation of insectivorous taxa, prioritizing ecological diversity over statistical robustness. This approach enabled a more comprehensive assessment of AR exposure risk across species with different diets, foraging behaviors, and habitat preferences, thereby improving the ecological relevance of the findings.

Secondly, there are biogeographic constraints that affect interpretation. While the bird samples came from five of the eight main Canary Islands, offering a relatively broad regional scope, all the reptile samples (veiled chameleons) were limited to the island of Gran Canaria, and even within this island, they were restricted to a few municipalities. This discrepancy reflects the limited geographic distribution of *Chamaeleo calyptratus* in the archipelago, currently confined to a small area in Gran Canaria. The species was first detected in the wild in 2017 by the Canary Islands’ early warning system for invasive species, and since then, the invasion has remained geographically contained. Nevertheless, *C. calyptratus* has been present on the island long enough to act as a useful sentinel for long-term environmental exposure to anticoagulant rodenticides, particularly due to its insectivorous diet and terrestrial habits. This situation underscores the need to incorporate additional sentinel species with broader distributions across the archipelago to more accurately assess spatial variability in AR exposure.

Thirdly, a key limitation of the present study is the lack of direct analysis of invertebrate prey. Although our findings strongly suggest that secondary exposure to ARs may occur through the consumption of contaminated invertebrates, we were not able to test this pathway directly. The detection of SGAR residues in insectivorous wildlife is consistent with this hypothesis, but confirmation will require future studies that include the analysis of potential invertebrate vectors. Such studies would provide a crucial link in understanding the transmission of rodenticides through the terrestrial food web and further validate the role of invertebrates as vectors of toxicants.

Finally, statistical comparisons between birds and reptiles were deliberately avoided, given the taxonomic and ecological differences between the two groups. Their distinct metabolic rates, susceptibility to toxicants, and exposure pathways preclude meaningful quantitative comparisons. Therefore, the study’s strength lies not in inferential statistics but in the qualitative and descriptive evidence it provides.

Despite these limitations, this study presents several key strengths. Most notably, it offers robust evidence that second-generation anticoagulant rodenticides (SGARs) have penetrated the lowest trophic levels of the terrestrial food web in the Canary Islands. The detection of SGARs in nearly 80% of the insectivorous reptiles and 40% of the non-raptorial insectivorous birds underscores the pervasive environmental contamination by these compounds. By focusing on species at the base of the food chain, the study sheds light on a largely overlooked exposure pathway—namely, secondary exposure via invertebrate prey—which may be of particular concern in insular ecosystems characterized by high endemism and ecological sensitivity.

In this context, our findings contribute to the growing body of evidence that highlights the widespread and insidious presence of SGARs in island environments. They emphasize the need to expand biomonitoring efforts beyond apex predators and to consider the full spectrum of ecological interactions when assessing the risks of rodenticide use in biodiversity hotspots.

## 5. Conclusions

Tracking the movement of anticoagulant rodenticides (ARs) through food webs is essential to assessing their ecological impact. Invertebrates occupy a foundational position in terrestrial trophic networks and serve as key prey for a variety of vertebrate species. This study provides valuable information on SGAR contamination in the lower trophic levels of the Canary Islands’ terrestrial ecosystems, with detections in 77.8% of sampled reptiles and 39.4% of non-raptorial insectivorous birds. The prevalence of low AR concentrations (<50 ng/g ww.) supports the hypothesis that secondary exposure via the ingestion of contaminated invertebrates is a likely pathway. Our findings highlight the potential of the veiled chameleon as a valuable sentinel for AR exposure in insular reptile communities. Additionally, the observed variation in exposure among birds suggests that habitat preferences and foraging strategies may modulate individual risk.

However, a key limitation of this study is the small sample size for some studied species and the absence of direct data on AR residues in invertebrate prey. Without empirical evidence of accumulation in these organisms, their role in trophic transfer remains unconfirmed. Future studies should include more individuals and prioritize the analysis of invertebrate taxa commonly consumed by insectivorous vertebrates to clarify their involvement in AR transmission and to enhance environmental risk assessments, particularly in insular ecosystems.

## Figures and Tables

**Table 1:** Descriptive statistics of brodifacoum (ng/g ww.) concentrations in *Chamaeleo calyptratus* specimens from Gran Canaria (n = 36).

Brodifacoum (ng/g ww.)								
n+ (%)	Mean Total	Mean Detected	Median Total	Median Detected	SD	Max	P25	P75
28 (77.8)	6.82	8.73	3.13	4.36	8.94	33.11	0.55	8.57

Values are presented for brodifacoum in detected individuals and for the total series, including mean, median, standard deviation (SD), maximum, and the 25th and 75th percentiles (P25, P75). All data are presented in ng/g ww.

**Table 2:** Descriptive statistics of ARs in non-raptor birds from the Canary Islands.

	Sum of ARs (ng/g ww.)					Brodifacoum (ng/g ww.)				
Species	n+ (%)	Mean (+) ± SD	Median (+)	Mean Total ± SD	Median Total	n+ (%)	Mean (+) ± SD	Median (+)	Mean Total ± SD	Median Total
Hoopoe (n = 5)	5 (100)	102.65 ± 195.18	26.24	102.65 ± 195.18	26.24	5 (100)	102.65 ± 195.18	26.24	102.65 ± 195.18	26.24
Stone-curlew (n = 44)	22 (50)	19.30 ± 49.85	4.79	9.65 ± 36.18	0.17	21 (47.8)	19.87 ± 50.97	4.86	9.48 ± 36.18	0
Houbara bustard (n = 22)	2 (9.1)	0.72 ± 0.09	0.72	0.07 ± 0.21	0	2 (9.1)	0.72 ± 0.09	0.72	0.07 ± 0.21	0
Blackbird (n = 18)	6 (33.3)	3.24 ± 4.64	1.65	1.08 ± 2.97	0	6 (33.3)	3.24 ± 4.64	1.65	1.08 ± 2.97	0
Woodpecker (n = 5)	1 (20)	3.16	3.16	0.63 ± 1.41	0	0	-	-	-	-
Common swift (n = 4)	3 (75)	5.89 ± 7.19	2.68	4.42 ± 6.57	1.78	2 (50)	1.73 ± 1.35	1.73	0.86 ± 1.26	0.39

Values are presented for the sum of detected compounds and for brodifacoum individually, including frequency of detection, mean, median, and standard deviation (SD), for positive individuals and for the total series.

## Data Availability

The data that support the findings of this study are available from the corresponding author upon reasonable request.

## Supplementary Materials

The following supporting information can be downloaded at: https://www.mdpi.com/article/10.3390/toxics13060505/s1, Table S1: Limits of detection (LODs) and quantification (LOQs) in ng/mL of the extraction method; Table S2: Descriptive table of *Chamaeleo calyptratus* specimens from Gran Canaria; Table S3: Dataset of non-raptor bird species from the Canary Islands.

## Abbreviations

The following abbreviations are used in this manuscript:

SGAR	Second-Generation Anticoagulant Rodenticide
FGAR	First-Generation Anticoagulant Rodenticide
AR	Anticoagulant Rodenticide
LOD	Limit of Detection
LOQ	Limit of Quantification
QCs	Quality Control
SERTOX	Toxicology Service
VKORC	Vitamin K Epoxide Reductase
SVL	Snout-vent length
SCIs	Sites of Community Importance
SPAs	Special Protection Areas for Birds
RSD	Relative Standard Deviation
P-IS	Internal Standard Procedural
ACN	Acetonitrile
FA	Formic Acid
