# Supplementary material for: Widespread Contamination by Anticoagulant Rodenticides in Insectivorous Wildlife from the Canary Islands: Exploring Alternative Routes of Exposure
Source: Toxics. 2025 Jun 15;13(6):505. doi: 10.3390/toxics13060505 (PMC12197510; doi:10.3390/toxics13060505)
Supplement: Supplementary file 1 [file toxics-13-00505-s001.zip › toxics-3660797-supplementary.pdf]

Supplementary Table S1. Limits of detection (LODs) and quantification (LOQs) in ng/mL of the extraction method. [75]

**Compound**	**Group**	**LOQ**	**LOD**
Brodifacoum	Rodenticides	0.4	0.34
Bromadiolone	Rodenticides	0.4	0.34
Chlorophacinone	Rodenticides	8	6.8
Coumachlor	Rodenticides	0.8	0.68
Coumatetralyl	Rodenticides	1.6	1.36
Diphacinone	Rodenticides	8	6.8
Difenacoum	Rodenticides	0.8	0.68
Difethialone	Rodenticides	1.6	1.36
Flocoumafen	Rodenticides	0.4	0.34
Warfarin	Rodenticides	0.8	0.68

[75] Rial-Berriel, C.; Acosta-Dacal, A.; Zumbado, M.; Alberto Henríquez-Hernández, L.; Rodríguez-Hernández, Á.; Macías-Montes, A.; Boada, L.D.; Del Mar Travieso-Aja, M.; Cruz, B.M.; Luzardo, O.P. A Method Scope Extension for the Simultaneous Analysis of POPs, Current-Use and Banned Pesticides, Rodenticides, and Pharmaceuticals in Liver. Application to Food Safety and Biomonitoring. *Toxics* **2021**, *9*, 238, doi:10.3390/toxics9100238.

Supplementary Table S2. Descriptive table of *Chamaeleo calyptratus* specimens from Gran Canaria.

ID	Island	Municipality	Year	Sex	SVL (cm)	Weight (g)	Brodifacoum (ng/g ww)	Bromadiolone (ng/g ww)	∑ SGARs (ng/g ww)
CAM001	Gran Canaria	Arucas	2023	Male	19	133	3.78		3.78
CAM002	Gran Canaria	Arucas	2023	Female	13.5	80	8.01		8.01
CAM003	Gran Canaria	Arucas	2023	Male	16.4	111	11.02		11.02
CAM004	Gran Canaria	Arucas	2023	Male	12.6	96			-
CAM005	Gran Canaria	Arucas	2023	Female	14.3	92			-
CAM006	Gran Canaria	Arucas	2023	Male	15.8	88			-
CAM007	Gran Canaria	Arucas	2023	Male	15	89	0.96		0.96
CAM008	Gran Canaria	Arucas	2023	Female	11.8	115	3.23		3.23
CAM009	Gran Canaria	Arucas	2023	Male	15.8	98	1.77		1.77
CAM010	Gran Canaria	Arucas	2023	Male	14.9	102	8.4		8.4
CAM011	Gran Canaria	Arucas	2023	Male	11.9	49	0.63		0.63
CAM012	Gran Canaria	Arucas	2023	Female	12.2	53	0.58		0.58
CAM013	Gran Canaria	Arucas	2023	Male	17	103	0.8		0.8
CAM014	Gran Canaria	Arucas	2024	Female	13.2	-	9.06		9.06
CAM015	Gran Canaria	Arucas	2024	Male	16.7	125	1.5		1.5
CAM016	Gran Canaria	Arucas	2024	Male	14.7	87	28.27		28.27
CAM017	Gran Canaria	Arucas	2024	Male	13.4	74	3.51		3.51
CAM018	Gran Canaria	Arucas	2024	Female	11.4	60	0.47		0.47
CAM019	Gran Canaria	Arucas	2024	Female	-	-	3.03		3.03
CAM020	Gran Canaria	Arucas	2024	Female	15	107	33.11		33.11
CAM021	Gran Canaria	Arucas	2024	Unknown	5	10	2.36		2.36
CAM022	Gran Canaria	Arucas	2024	Female	16.5	88	20.89	0.47	21.36
CAM023	Gran Canaria	Arucas	2024	Male	16	112			-
CAM024	Gran Canaria	Arucas	2024	Male	14.5	86	24.94		24.94
CAM025	Gran Canaria	Arucas	2024	Male	14	74	11.67		11.67
CAM026	Gran Canaria	Arucas	2024	Male	15	100			-
CAM027	Gran Canaria	Arucas	2024	Unknown	9	18			-
CAM028	Gran Canaria	Arucas	2024	Female	10	45			-
CAM029	Gran Canaria	Arucas	2024	Female	14	103	14.71		14.71
CAM030	Gran Canaria	Arucas	2024	Female	14.5	118	24.21		24.21
CAM031	Gran Canaria	Arucas	2024	Male	12.5	49	3.98		3.98
CAM032	Gran Canaria	Arucas	2024	Female	12	42			-
CAM033	Gran Canaria	Arucas	2024	Male	18	200	4.74		4.74
CAM034	Gran Canaria	Arucas	2024	Male	18.3	160	7.97		7.97
CAM035	Gran Canaria	Arucas	2024	Male	12	55	2.62		2.62
CAM036	Gran Canaria	Arucas	2024	Female	11	44	8.33		8.33

Data include municipality and island of origin, year of collection, sex, snout–vent length (SVL), body weight (g) and detected concentrations (ng/g liver ww) of each compound with total anticoagulant rodenticides (∑ARs) in *Chamaeleo calyptratus* specimens (n = 36) collected in Gran Canaria.

Supplementary Table S3. Dataset of non-raptor bird species from Canary Islands.

Species	Island	Year	Brodifacoum (ng/g ww)	Bromadiolone (ng/g ww)	Difenacoum (ng/g ww)	Difethialone (ng/g ww)	ΣSGARs (ng/g ww)
Hoopoe	Lanzarote	2021	451.2				451.2
Hoopoe	Tenerife	2021	26.24				26.24
Hoopoe	Tenerife	2022	27.69				27.69
Hoopoe	-	2022	3.78				3.78
Hoopoe	Gran Canaria	2023	4.36				4.36
Stone-curlew	Gran Canaria	2021	11.4				11.4
Stone-curlew	Tenerife	2021	6.85				6.85
Stone-curlew	Lanzarote	2021	4.72				4.72
Stone-curlew	Lanzarote	2021	10.3				10.3
Stone-curlew	Lanzarote	2021					-
Stone-curlew	Lanzarote	2021					-
Stone-curlew	Gran Canaria	2021					-
Stone-curlew	Gran Canaria	2021					-
Stone-curlew	Lanzarote	2021	7.42				7.42
Stone-curlew	Lanzarote	2021					-
Stone-curlew	Gran Canaria	2021					-
Stone-curlew	Lanzarote	2024					-
Stone-curlew	Lanzarote	2022	1.74				1.74
Stone-curlew	Gran Canaria	2022					-
Stone-curlew	Gran Canaria	2022	50.25				50.25
Stone-curlew	Gran Canaria	2022	1.46				1.46
Stone-curlew	Lanzarote	2022					-
Stone-curlew	-	2022	235.47				235.47
Stone-curlew	-	2022		1.35			1.35
Stone-curlew	-	2022					-
Stone-curlew	-	2022	36.83				36.83
Stone-curlew	Lanzarote	2022					-
Stone-curlew	Gran Canaria	2022	9.59	2.1		2.94	14.63
Stone-curlew	-	2023	0.73				0.73
Stone-curlew	Fuerteventura	2023					-
Stone-curlew	Fuerteventura	2023					-
Stone-curlew	Gran Canaria	2023	0.35*				0.35
Stone-curlew	Gran Canaria	2023					-
Stone-curlew	Gran Canaria	2023					-
Stone-curlew	Fuerteventura	2023	0.38*				0.38
Stone-curlew	Lanzarote	2024	1.21				1.21
Stone-curlew	Lanzarote	2024	1.7				1.7
Stone-curlew	-	2024	1.14				1.14
Stone-curlew	Gran Canaria	2024					-
Stone-curlew	Gran Canaria	2024	2.5	1.02			3.52
Stone-curlew	Fuerteventura	2024					-
Stone-curlew	Gran Canaria	2024	18.34				18.34
Stone-curlew	Gran Canaria	2024	10				10
Stone-curlew	-	2023					-
Stone-curlew	Gran Canaria	2023	4.86				4.86

Species	Island	Year	Brodifacoum (ng/g ww)	Bromadiolone (ng/g ww)	Difenacoum (ng/g ww)	Difethialone (ng/g ww)	ΣSGARs (ng/g ww)
Stone-curlew	Fuerteventura	2023					-
Stone-curlew	Lanzarote	2023					-
Stone-curlew	Gran Canaria	2023					-
Stone-curlew	Lanzarote	2023					-
Houbara bustard	Lanzarote	2021					-
Houbara bustard	Lanzarote	2021					-
Houbara bustard	Lanzarote	2021					-
Houbara bustard	Lanzarote	2021					-
Houbara bustard	Lanzarote	2021					-
Houbara bustard	Lanzarote	2021					-
Houbara bustard	Lanzarote	2021					-
Houbara bustard	Lanzarote	2021					-
Houbara bustard	Lanzarote	2021	0.78				0.78
Houbara bustard	Lanzarote	2021					-
Houbara bustard	Lanzarote	2021					-
Houbara bustard	Lanzarote	2024	0.65				0.65
Houbara bustard	Lanzarote	2021					-
Houbara bustard	Lanzarote	2022					-
Houbara bustard	Fuerteventura	2023					-
Houbara bustard	Fuerteventura	2023					-
Houbara bustard	Lanzarote	2023					-
Houbara bustard	Lanzarote	2023					-
Houbara bustard	Lanzarote	2024					-
Houbara bustard	Fuerteventura	2024					-
Houbara bustard	Lanzarote	2022					-
Houbara bustard	Lanzarote	2023					-
Blackbird	Gran Canaria	2021					-

Species	Island	Year	Brodifacoum (ng/g ww)	Bromadiolone (ng/g ww)	Difenacoum (ng/g ww)	Difethialone (ng/g ww)	ΣSGARs (ng/g ww)
Blackbird	Gran Canaria	2021					-
Blackbird	Gran Canaria	2021					-
Blackbird	Gran Canaria	2021					-
Blackbird	Gran Canaria	2021					-
Blackbird	Gran Canaria	2021					-
Blackbird	Gran Canaria	2021					-
Blackbird	Gran Canaria	2021					-
Blackbird	Gran Canaria	2024	0.9				0.9
Blackbird	Gran Canaria	2024	1.81				1.81
Blackbird	Tenerife	2022					-
Blackbird	Gran Canaria	2022	12.66				12.66
Blackbird	Gran Canaria	2024	0.75				0.75
Blackbird	Gran Canaria	2023	1.84				1.84
Blackbird	Gran Canaria	2023					-
Blackbird	Gran Canaria	2022					-
Blackbird	Gran Canaria	2022					-
Blackbird	La Palma	2023	1.49				1.49
Woodpecker	Gran Canaria	2021					-
Woodpecker	Tenerife	2021					-
Woodpecker	Tenerife	2021			3.16		3.16
Woodpecker	Gran Canaria	2021					-
Woodpecker	Tenerife	2021					-
Common swift	Lanzarote	2021					-
Common swift	Gran Canaria	2024	26.83				26.83
Common swift	Gran Canaria	2024	0.77	1.46		11.9	14.13
Common swift	Gran Canaria	2024		0.87			0.87

Data include the information regarding island of origin, year of collection, and detected compounds, as well as the ΣSGARs in ng/g ww for each bird. (*: Values between LOD-LOQ)
